# A novel test of the GTO implementation support intervention in low resource settings: Year 1 findings and challenges

**DOI:** 10.1186/1748-5908-10-S1-A34

**Published:** 2015-08-20

**Authors:** Matthew Chinman, Joie Acosta, Patricia Ebener, Patrick S Malone, Mary Slaughter

**Affiliations:** 1 RAND Health, RAND Corporation, Pittsburgh, PA 15213, USA; 2 VISN 4 MIRECC, VA Pittsburgh Healthcare System, Pittsburgh, PA 15240, USA; 3 RAND Health, RAND Corporation, Arlington, VA 22202-5050, USA; 4 RAND Health, RAND Corporation, Santa Monica, CA 90401-3208, USA; 5 Malone Quantitative, Durham, NC 27704, USA

## Introduction

Implementation research is expanding because Evidence-Based Programs (EBPs) are not adopted in many medical domains. However, rigorous implementation research is needed in nonclinical, community-based settings, which often have low capacity that undermines implementation quality and outcomes. This presentation describes Enhancing Quality Interventions Promoting Healthy Sexuality, a 5-year, cluster Hybrid Implementation RCT (Type II) testing how well a community-based setting (Boys & Girls Clubs, BGCs) conducts an EBP called Making Proud Choices (MPC) that aims to prevent teen pregnancy and sexually transmitted infections, with and without an implementation support intervention called Getting To Outcomes (GTO). GTO is a 10-step model of program operation grounded in implementation theory and an intervention of written tools, training and bi-weekly onsite technical assistance.

## Methods

The trial compares 16 BGCs implementing the MPC program for two years, in the fashion typical of community settings, with 16 BGCs implementing MPC augmented with GTO. Capacity to carry out key program tasks prescribed by GTO's 10 steps is measured using ratings made from a standardized, structured interview with program personnel at all 32 BGC sites after each year of implementation. Fidelity of MPC is assessed at all sites by observer ratings of adherence. Youth sexual health outcomes (knowledge, attitudes, and behaviors around condoms and sex) are assessed via surveys before, immediately following, and 6-months after MPC.

## Findings

After one year, GTO sites had significantly higher capacity ratings. Between groups, MPC fidelity ratings were similar and youth improved similarly on condom attitudes and behavior and sexual knowledge. Conclusions: This study is the first that assesses an implementation intervention's impact on capacity, implementation quality, and individual outcomes simultaneously and in both study conditions. GTO improved capacity, but fidelity and sex outcomes improved similarly. Methodological challenges' impact on these early results will be presented and potential solutions offered.

The study is novel in that it tests the impact of an implementation support intervention in low capacity settings and assesses capacity, implementation quality, and individual outcomes simultaneously and in both intervention and control conditions. Also, this test of GTO's implementation intervention has revealed a number of methodological challenges that are not often discussed in the implementation literature, but need to be addressed.

